# Molecular signatures of neutrophil extracellular traps in human visceral leishmaniasis

**DOI:** 10.1186/s13071-017-2222-5

**Published:** 2017-06-06

**Authors:** Luiz Gustavo Gardinassi, Thiago S. DeSouza-Vieira, Naila O. da Silva, Gustavo R. Garcia, Valéria M. Borges, Roseane N. S. Campos, Roque P. de Almeida, Isabel K. F. de Miranda Santos, Elvira M. Saraiva

**Affiliations:** 1 Departamento de Bioquímica e Imunologia, Faculdade de Medicina de Ribeirão Preto, Universidade de São Paulo, Ribeirão Preto, SP Brazil; 2 Departamento de Imunologia, Instituto de Microbiologia Paulo de Góes, Universidade Federal do Rio de Janeiro, Rio de Janeiro, RJ Brazil; 3 Instituto Gonçalo Moniz, Fundação Oswaldo Cruz (FIOCRUZ), Salvador, BA Brazil; 4 Departamento de Medicina, Hospital Universitário, Universidade Federal de Sergipe, Aracaju, SE Brazil

**Keywords:** Neutrophil extracellular traps, NETosis, *Leishmania infantum*, Visceral leishmaniasis, Asymptomatic infection

## Abstract

**Background:**

Infections with parasites of the *Leishmania donovani* complex result in clinical outcomes that range from asymptomatic infection to severe and fatal visceral leishmaniasis (VL). Neutrophils are major players of the immune response against *Leishmania*, but their contribution to distinct states of infection is unknown. Gene expression data suggest the activation of the NETosis pathway during human visceral leishmaniasis. Thus, we conducted an exploratory study to evaluate NET-related molecules in retrospective sera from VL patients, asymptomatic individuals and uninfected endemic controls.

**Results:**

We demonstrate that VL patients and asymptomatic individuals exhibit differential regulation of molecules associated with neutrophil extracellular traps (NET). These differences were observed at the transcriptional level of genes encoding NET-associated proteins; in quantifications of cell free DNA and metalloproteinase 9; and in enzymatic activity of DNAse and elastase. Moreover, multivariate analysis resulted in class-specific signatures, and ROC curves demonstrate the ability of these molecules in discriminating asymptomatic infection from uninfected controls.

**Conclusion:**

Molecules that are associated with NETs are differentially regulated between distinct states of infection with *L*. *infantum*, suggesting that NETs might have distinct roles depending on the clinical status of infection. Although unlikely to be exclusive for VL, these signatures can be useful to better characterize asymptomatic infections in endemic regions of this disease.

**Electronic supplementary material:**

The online version of this article (doi:10.1186/s13071-017-2222-5) contains supplementary material, which is available to authorized users.

## Background

Visceral leishmaniasis (VL) is a life-threatening disease caused by infections with the protozoan parasites *Leishmania donovani* and *L*. *infantum*. It is estimated that over 20,000 deaths occur annually due to VL, however, most infections remain asymptomatic [1]. The prognosis of asymptomatic infection and its role in transmission are unknown; furthermore, the mechanisms underlying either active VL or asymptomatic infections have not been fully elucidated. An effective immune response should account for a favorable outcome after infection, thus asymptomatic infections can provide important insights towards understanding host responses during infections with *Leishmania* [2]. Neutropenia is an independent predictor of risk of dying from VL [3], whereby systemic inflammation plays a major role. These findings indicate that neutrophil activity may be involved in the distinct states of infection; however, the mechanisms linking neutrophils to active VL or asymptomatic infection are unknown.

Neutrophils rapidly migrate and infiltrate into sites of infection and inflammation. They are important sources of anti-microbial effector molecules involved in host defense, some of which cause tissue damage. Microbicidal activity of neutrophils is exerted by proteolytic enzymes, reactive oxygen species and neutrophil extracellular traps (NETs), which exhibit critical roles during infections with a wide range of pathogens. The release of NETs emerged as an important process of host response to extracellular bacteria and fungi [4, 5]; we demonstrated that *Leishmania* also trigger this response [6]: *L*. *donovani* and *L*. *infantum* induce release of NETs by human neutrophils, but they are capable of evading killing through expression of lipophosphoglycan or 3′-nucleotidase/nuclease activity, respectively [7, 8]. In spite of this knowledge, the impact of NETosis during distinct outcomes of human infections with *Leishmania* remains unexplored.

Here, we evaluated gene expression data from blood of VL patients, asymptomatic individuals and uninfected controls; we observed significant differences between these groups in the modulation of genes encoding NET-associated proteins. We also quantified levels of cell-free DNA and metalloproteinase 9; or measured the enzymatic activity of DNAse, elastase and myeloperoxidase in retrospective sera from distinct states of infection with *L*. *infantum*. We identified profiles that suggest a differential regulation of NETosis pathway between states of infection with *L*. *infantum*. Importantly, these signatures point towards novel perspectives for characterization of asymptomatic infections in endemic areas for VL.

## Methods

### Study groups

In this study, we used retrospective serum samples collected from patients with uncomplicated VL at University Hospital, UFS, Aracaju-SE, Brazil (*n* = 35; male to female ratio = 1.9; mean age ± standard deviation (SD) = 31.5 ± 18.3 years), and stored at -80 °C. All samples were collected before treatment of patients. Diagnosis was confirmed by identification of *Leishmania* amastigotes in Giemsa-stained smears, positive culture on NNN media of bone marrow aspirate or rK39 positive serology. Retrospective serum samples of healthy individuals living in the same areas were also used. Individuals were tested for delayed type hypersensitivity (reflecting cellular immune response) and IgG reactivity (reflecting humoral immune response) to leishmanial antigens as described previously [9]. Only subjects exhibiting positive reactions to both tests were considered to be asymptomatically infected (*n* = 28; male to female ratio = 1.1; mean age ± SD = 31.5 ± 15.0 years). Controls included individuals who exhibited negative reactions (*n* = 25; male to female ratio = 0.9; mean age ± SD = 30.6 ± 18.1 years). The groups did not present significant differences with respect to age (tested with ANOVA) or sex distribution (tested with Chi-square test).

### Whole blood transcriptional analyses

Blood collection, RNA extraction, hybridization, scanning and raw data pre-processing pipeline were described previously [10]. Differential gene expressions between distinct groups were identified with linear models and moderated *t*-statistic. Differentially expressed genes (DEGs) were evaluated with GeneGo MetaCore (Thomson Reuters, NY, USA). *P-*values were adjusted with Benjamini-Hochberg, and DEGs or pathway over-representation were identified by a FDR or *q* < 0.05. Gene set enrichment analysis (GSEA) was performed in pre-ranked list mode with 1000 permutations and weighted enrichment statistic.

### Measurement of extracellular dsDNA

Serum was diluted 1:4 in TE (Tris-HCl 10 mM, pH 8,0; EDTA 1 mM) and quantified with Quant-iT™ PicoGreen® dsDNA Assay kit (Thermo Fisher Scientific, Waltham, USA) as reported previously [6].

### DNAse activity assay

NET-DNA enriched supernatants were obtained as described previously [5]. Serum was diluted 1:10 and further incubated with 7 μg/ml of NET-DNA enriched supernatants and DNAse cofactors (500 μM CaCl_2_; 5 mM MgCl_2_) at 35 °C and 5% CO_2_. After 5 h, DNAse activity was calculated as the percentage of NET-DNA degradation using Quant-iT™ PicoGreen® dsDNA Assay kit (Thermo Fisher Scientific, Waltham, USA). Purified DNAse-I (2 units; Promega, Fitchburg, USA) was used as a positive control of DNAse activity. Untreated NET-DNA supernatants were used as reference for calculations.

### Elastase, myeloperoxidase (MPO) and metalloproteinase 9 (MMP-9) assays

Serum was diluted 1:4 in buffer (50 mM HEPES, 100 mM NaCl and 0.01% Triton X-100) and elastase activity was measured with the fluorogenic substrate N-Methoxysuccinyl-Ala-Ala-Pro-Val-7-amido-4-methylcoumarin (Sigma-Aldrich, St. Louis, USA) according to manufacturer’s instructions. Serum was diluted 1:5 in PBS 0.5% Hexadecyltrimethylammonium bromide (5 mg/ml) and peroxidase activity was measured using 3,3′,5,5′-Tetramethylbenzidine (Sigma) after incubation for 1 h at 35 °C. Absorbance was detected on a SpectraMax® Paradigm® microplate reader at 630 nm (Molecular Devices, California, USA). Quantification of serum MMP-9 was performed with Human MMP-9 Quantikine ELISA kit (R&D Systems, Minneapolis, USA) according to instructions provided by the supplier.

### Statistical analysis

One-way ANOVA with Bonferroni’s or Kruskal-Wallis with Dunn’s multiple comparison tests were applied to evaluate differences among independent groups. Spearman’s rank correlation was used to assess non-parametric associations. *P-*values less than 0.05 were considered significant. Hierarchical clustering was performed with Euclidian distance for metric calculations and the complete linkage method. Receiver Operating Characteristic (ROC) curves were generated with the online analysis platform MetaboAnalyst 3.0 [11], with PLS-DA as a classification method and t-statistic as a ranking metric.

## Results and discussion

**Fig. 1:**
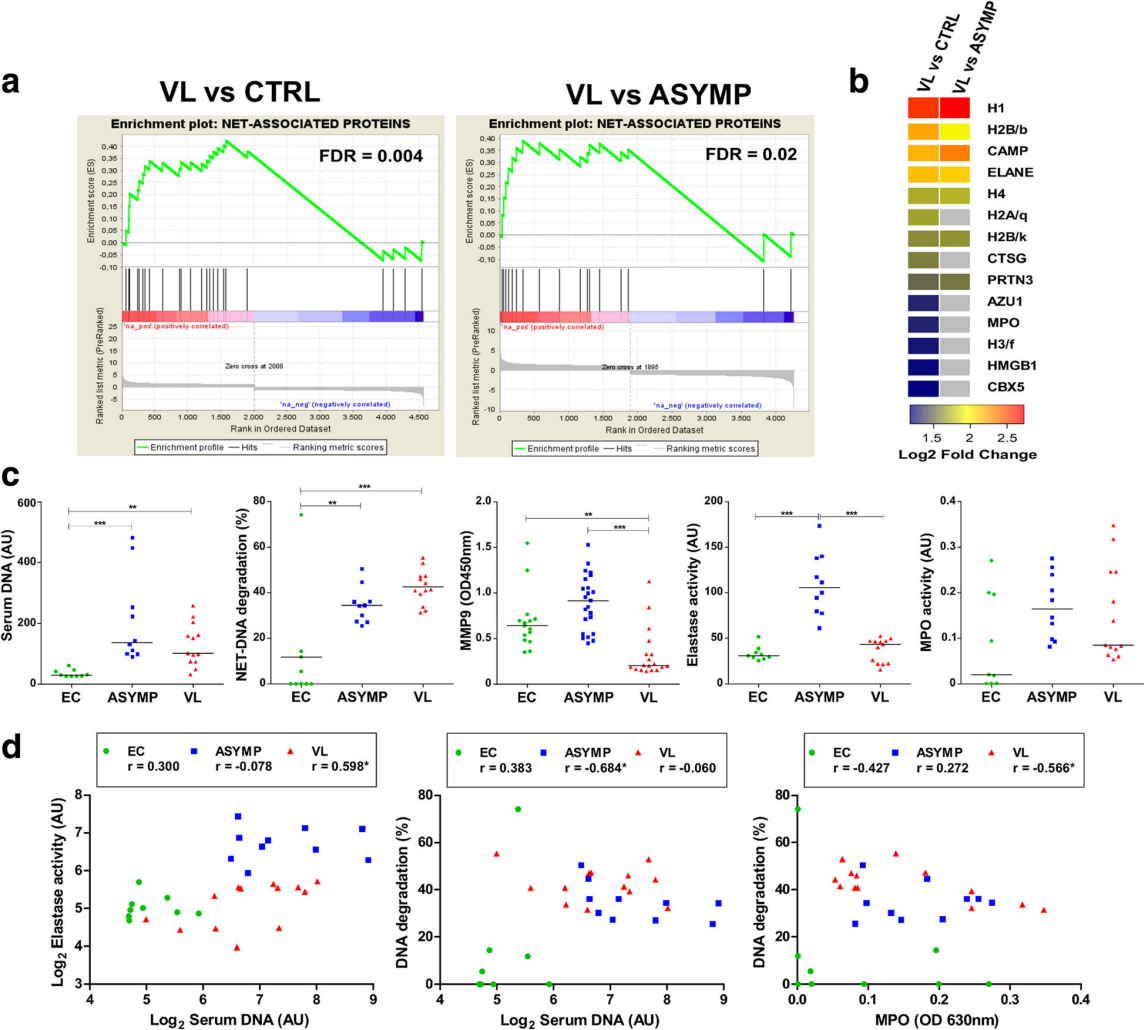
Changes in transcriptional and functional activity of indicators of NETosis depend on the outcome of infection with *Leishmania infantum*. **a** Gene set enrichment analysis (GSEA) showing statistically significant enrichment of differentially expressed genes from VL patients in the “NET-associated proteins” gene-set (FDR = false discovery rate). **b** Heat map displaying log2 fold changes of genes in the “core enrichment” identified by GSEA. **c** Levels of serum DNA and matrix metalloproteinase 9 (MMP-9) or activity of DNAse, elastase and myeloperoxidase (MPO) are shown for endemic controls (EC; *green circles*), asymptomatic individuals (ASYMP; *blue squares*) and VL patients (VL; *red triangles*). Data were analyzed with ANOVA with Bonferroni’s or Kruskal-Wallis with Dunn’s multiple comparison test. Lines represent mean or median values. **d** Correlations between serum DNA, elastase activity, DNAse activity and MPO activity. Spearman’s rank correlation was used to assess significant associations for EC (*n* = 9), ASYMP (*n* = 10) and VL (*n* = 13). Significance is given *P < 0.05, **P < 0.01 and ***P < 0.001

We recently demonstrated that, compared to asymptomatic individuals or controls, microarray data expression from whole blood of VL patients was associated with the perturbation of several immune related pathways annotated in the GeneGO Metacore database [10]. Differentially expressed genes (DEGs, *q* < 0.05) from VL patients were enriched in the “NETosis in SLE” pathway (Additional file 1: Figure S1). To further investigate this finding, we generated a gene-set termed “NET-associated proteins”, based on genes coding for proteins shown to be enriched in NETs released from human neutrophils with mass spectrometry [5, 12]. GSEA with DEGs from VL patients pre-ranked by expression fold-change resulted in significant associations between the transcriptional profile of VL patients and the “NET-associated proteins” gene-set (Fig. 1a, NES = 2.20, *P* = 0.004 for VL and uninfected controls; NES = 1.80, *P* = 0.02 for VL and asymptomatic infection). Indeed, the majority of genes coding for proteins associated with NETs were upregulated in VL patients (Fig. 1a, b). However, compared to uninfected controls, VL patients exhibited more DEGs in the core enrichment than compared to asymptomatic individuals (Fig. 1b). Due the inflammatory nature of NETs, it is unlikely that this activity of the NETosis pathway is exclusive for *Leishmania* infection. Thus, we evaluated publicly available gene expression profiles (GEO database) of patients infected with *Mycobacterium tuberculosis* (TB), individuals with latent infection with *M*. *tuberculosis* (LTB) and uninfected controls [13]. Of interest, we also identified differential regulation of “NET-associated proteins” gene set among the distinct states of infection with *M*. *tuberculosis* (Additional file 2: Figure S2; NES = 2.77, *P* < 0.0001 for TB and uninfected controls; NES = 2.06, *P* < 0.0001 for TB and LTB). Taken together, those findings suggest that expression profiles might reflect the activity of the NETosis pathway during infections, and might be differentially regulated between active disease and asymptomatic status as demonstrated for other immune compartments such as antibody profiles in tuberculosis [14] and VL [9], and transcriptional activity of type I interferon responses in VL [10].

To investigate further, we screened sera for known components of NETs in a retrospective cohort of subjects presenting with distinct statuses of infection with *L*. *infantum*. We quantified levels of cell free DNA and matrix metalloproteinase 9 (MMP-9), and measured the enzymatic activity of DNAse, elastase and myeloperoxidase (MPO). Compared to uninfected controls, levels of DNA were elevated in sera from VL patients and asymptomatic individuals (Fig. 1c, *H* = 18.43, *df* = 2, *P <* 0.0001). The degradation of NET-DNA, reflecting the enzymatic activity of DNAse, was also elevated in sera from VL patients and asymptomatic controls (Fig. 1c, *H* = 13.76, *df* = 2, *P* = 0.001). In contrast, the levels of MMP-9 were reduced in VL patients compared to both asymptomatic and uninfected controls (Fig. 1c, *H* = 25.15, *df* = 2, *P* < 0.0001), which did not differ between these two groups, as reported [15]. Surprisingly, the enzymatic activity of elastase was elevated in asymptomatic individuals, compared to both VL patients and uninfected controls (Fig. 1c, *H* = 20.12, *df* = 2, *P* < 0.0001), but VL and uninfected controls did not differ. Although a tendency for increased activity of MPO was observed for sera of asymptomatic individuals, there was not significant difference between the groups. Correlation analysis revealed a positive association between levels of DNA and elastase activity in sera from VL patients (Fig. 1d, *r* = 0.5, *P* = 0.03). Of note, levels of DNA in sera from asymptomatic individuals correlated negatively with activity of DNAse (Fig. 1d, *r* = -0.6, *P =* 0.03). Moreover, activity of DNAse and MPO were negatively correlated for VL patients (*r* = -0.5, *P* = 0.04). The lack of correlations between NET-associated molecules should be considered suggestive that the activity of those molecules might have a different origin than neutrophils. However, we speculate that the constitution and/or activity of the NETosis pathway could depend on the state of infection, as different stimulus can impact NET protein composition [16]. Whether the differential regulation between those molecules is indeed resulting from the release of NETs still needs detailed investigation. In this context, recent work demonstrated that VL patients present elevated levels of plasma arginase, MPO and elastase, which return to baseline levels after treatment, however asymptomatic infections were not analyzed [17]. We evaluated chronically infected individuals that should be carrying intracellular forms of *Leishmania*, raising questions about of the role of extracellular traps in asymptomatically infected and diseased subjects. One explanation could be that when amastigotes emerge from their niches to invade other cells they become susceptible to the activity of NETs. Recently, we reported an intimate association of extracellular amastigotes with NETs in cutaneous leishmaniasis lesions [18]. The spontaneous release of NETs plays an important role in several inflammatory diseases [16], giving another perspective to NETs in infections with *L*. *infantum*: spontaneous release of NETs during chronic VL could induce and/or maintain a systemic inflammatory response, contributing to the pathology of the disease. In a previous study, neutrophils from VL patients exhibited impaired release of NETs during stimulations in vitro [17]. However, the inflammatory environment to which neutrophils are submitted during the course of an infection has profound effects on neutrophils functions [19], and needs to be accounted for. Our study was limited to retrospective sera samples collected previously [9], but our data provide a proof of principle that the NETosis pathway can be important and should be investigated with more details in VL and asymptomatic infections. While neutrophil counts might also influence the quantification of the molecules evaluated herein, we highlight the fact that despite reduced proportions of granulocytes, VL patients still exhibited high activity of the NETosis pathway at the transcriptional level [10].

**Fig. 2:**
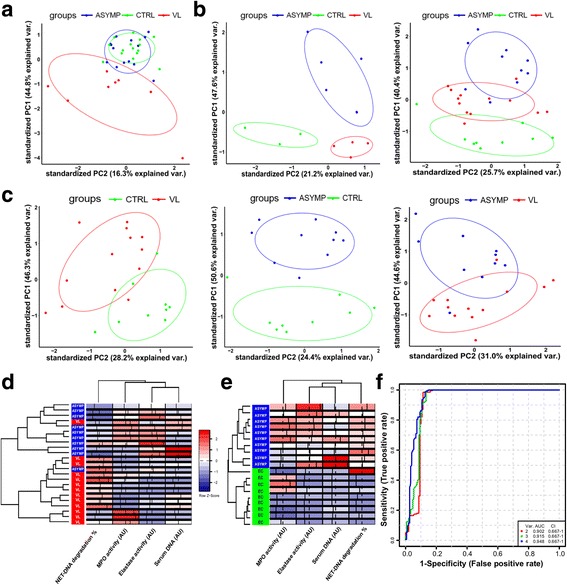
Indicators of NETosis compose biosignatures that discriminate distinct states of infection with *Leishmania infantum*. **a** Principal components analysis (PCA) based on expression data from genes included in the “NET-associated proteins” gene-set for endemic controls (EC; *green*), asymptomatic individuals (ASYMP; *blue*) and VL patients (VL; *red*). **b** PCA based on serum indicators of NETosis. Left-panel included measurements for levels of serum DNA and metalloproteinase 9 (MMP-9); and activity of DNAse, elastase and myeloperoxidase (MPO) from serum of EC (*n* = 3), ASYMP (*n* = 5) and VL (*n* = 4). Right-panel included measurements for levels of serum DNA; and activity of DNAse, elastase and myeloperoxidase (MPO) from serum of EC (*n* = 9), ASYMP (*n* = 10) and VL (*n* = 13). **c** PCA based on serum indicators of NETosis, excluding measurements for MMP-9. Left-panel depicts analysis for EC (*n* = 9) and VL (*n* = 13). Middle-panel depicts analysis for EC (*n* = 9) and ASYMP (*n* = 10). Right-panel depicts analysis for ASYMP (*n* = 10) and VL (*n* = 13). **d** Unsupervised hierarchical clustering of serum indicators of NETosis, depicted for ASYMP (*n* = 10) and VL (*n* = 13) including activity of DNAse (NET-DNA degradation %); activity of myeloperoxidase (MPO); activity of elastase and serum DNA. **e** Unsupervised hierarchical clustering of serum indicators of NETosis, depicted for ASYMP (*n* = 10) and EC (*n* = 9) including activity of DNAse (NET-DNA degradation %); activity of myeloperoxidase (MPO); activity of elastase and serum DNA. Analysis was performed with Euclidian distance for metric calculations and the complete linkage method. **f** ROC curves comparing three predictive models using 2, 3 or 4 features between asymptomatic individuals and endemic controls

Next, we conducted principal components analysis (PCA) to identify profiles of expression data of genes coding for NET-associated proteins or of serum indicators of NETosis from the groups of study. We observed a clear clustering pattern in expression data from VL patients and, as expected, those of asymptomatic individuals and uninfected controls were not distinguishable (Fig. 2a). Unfortunately, we were unable to perform all the serum assays for each of the subjects enrolled in the study, however, for a subset of individuals the analysis of principal components with indicators of NETosis revealed a successful segregation of subjects from distinct states of infection (Fig. 2b, left panel). Therefore, to gain power in the analysis by increasing the number of individuals from each group, we excluded measurements from MMP-9 and again found a consistent clustering pattern among subjects of specific groups (Fig. 2b, right panel). We next performed PCA based on peer-comparisons between the groups of study that confirmed distinct profiles of serum indicators of NETosis (Fig. 2c). To characterize the contribution of those molecules to distinct profiles between asymptomatic infection and active VL, we performed an unsupervised hierarchical clustering that retrieved biosignatures with the ability to discriminate between states of infection (Fig. 2d). Of note, asymptomatic individuals present an overall increase in levels of serum DNA and activity of MPO and elastase, while VL patients exhibit a strong signature of the activity of DNAse (Fig. 2d). Clustering of subjects in distinct groups, in addition to their original classification, suggests that the molecular signatures identified herein could also help in the design of prognosis scores, leading to a faster identification of individuals prone to develop VL and facilitate planning of treatments. For that purpose, longitudinal studies following asymptomatic carriers during a substantial period of time after infection might be useful to understand the relationship between molecular signatures of NETs and turnover of clinical status.

More striking was the observation that signatures composed by NET-related molecules distinguish asymptomatic individuals from uninfected controls (Fig. 2e). Most biomarkers of infection with *Leishmania* and diagnostic assays have been evaluated for their capacity to detect clinical disease, whereas the absence of robust markers for asymptomatic infection remains a problem [2]. Identification of these individuals is time consuming, expensive, and the availability of appropriate leishmanial antigens to performed DTH tests is often limited. The investigation of asymptomatic infections remains largely neglected, but can provide significant information to understand the immunobiology of VL [2]. NETosis is not an exclusive process for infections with *Leishmania*, and our data do not point for biomarkers of active VL. Moreover, an ideal biomarker exhibits unique associations with a particular phenotype. However, these molecular signatures might contribute to a better characterization of asymptomatic infection. This can be particularly useful in settings where only one method of characterization is available in endemic regions for VL. To support this concept, ROC curves were generated to verify the predictive value of these molecules in distinguishing asymptomatic *L*. *infantum* infection from uninfected individuals (Fig. 2f). By comparing models with varying numbers of features, the model with the 4 features exhibit the largest area under the curve (AUC - 0.948) and highest predictive accuracy value of 90.6%. This multivariate approach might reflect better the activity of the NETosis pathway in comparison to univariate analyses. Nevertheless, further investigation with larger numbers of individuals, and longitudinal designs will be necessary to validate these findings.

## Conclusions

In summary, our study prompts a more comprehensive evaluation of NETs during distinct states of infection with *Leishmania*. The signatures identified herein raise the hypothesis that NETs can play distinct roles depending on the progression of the infection. Of importance, the predictive value of these signatures point for novel directions to better characterize asymptomatic infections in endemic areas for VL.

## Additional files

Additional file 1: Figure S1. “NETosis in SLE” pathway map generated with GeneGO Metacore. Colored asterisks indicate differentially expressed genes of VL patients compared to uninfected controls, where red indicates upregulated genes and blue indicates downregulated genes. See MetaCore website for detailed legend at https://portal.genego.com/legends/MetaCoreQuickReferenceGuide.pdf. (TIFF 2787 kb)

Additional file 2: Figure S2. Gene set enrichment analysis (GSEA) showing statistically significant enrichment of differentially expressed genes from patients infected with *Mycobacterium tuberculosis* in the “NET-associated proteins” gene-set (FDR = false discovery rate). *M*. *tuberculosis* patients (TB), uninfected controls (CTRL) and latent *M*. *tuberculosis* infection (LTB). (TIFF 785 kb)

## Abbreviations

ANOVA: Analysis of variance
DEGs: Differentially expressed genes
DTH: Delayed type hypersensitivity
FDR: False discovery rate
GSEA: Gene set enrichment analysis
MMP-9: Metalloproteinase 9
MPO: Myeloperoxidase
NETs: Neutrophil extracellular traps
NNN: Novy-MacNeal-Nicolle
PCA: Principal components analysis
SLE: Systemic lupus erythematosus
VL: Visceral leishmaniasis
