# Supplementary figures and images for: Molecular signatures of neutrophil extracellular traps in human visceral leishmaniasis
Source: Parasit Vectors. 2017 Jun 6;10:285. doi: 10.1186/s13071-017-2222-5 (PMC5460406; doi:10.1186/s13071-017-2222-5)

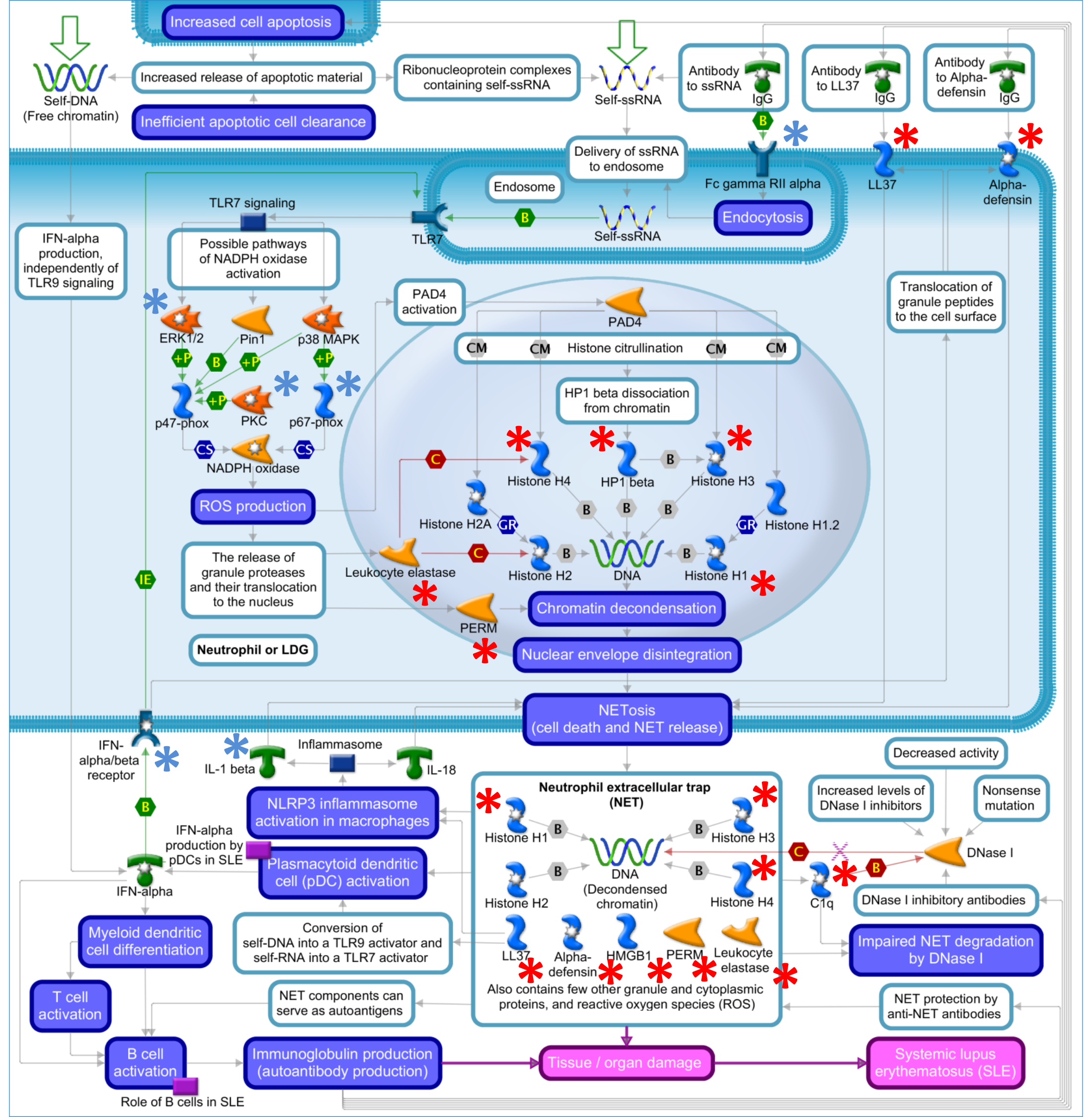

Supplement: Supplementary file 1 — “NETosis in SLE” pathway map generated with GeneGO Metacore. Colored asterisks indicate differentially expressed genes of VL patients compared to uninfected controls, where red indicates upregulated genes and blue indicates downregulated genes. See MetaCore website for detailed legend at https://portal.genego.com/legends/MetaCoreQuickReferenceGuide.pdf. (TIFF 2787 kb) [file 13071_2017_2222_MOESM1_ESM.tif]

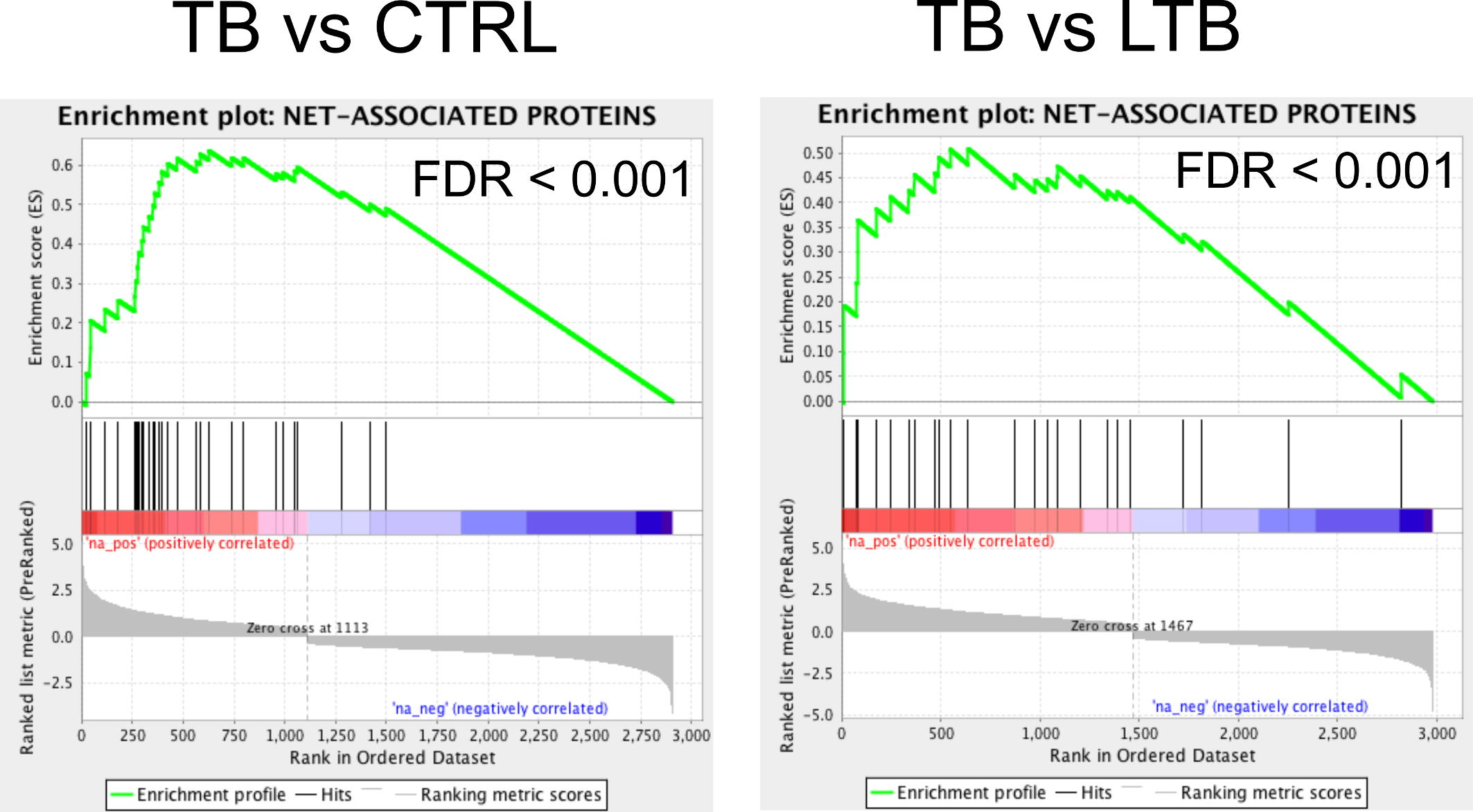

Supplement: Supplementary file 2 — Gene set enrichment analysis (GSEA) showing statistically significant enrichment of differentially expressed genes from patients infected with Mycobacterium tuberculosis in the “NET-associated proteins” gene-set (FDR = false discovery rate). M. tuberculosis patients (TB), uninfected controls (CTRL) and latent M. tuberculosis infection (LTB). (TIFF 785 kb) [file 13071_2017_2222_MOESM2_ESM.tif]
